# Crystal structure of methyl α-l-rhamno­pyranosyl-(1→2)-α-l-rhamno­pyran­oside monohydrate

**DOI:** 10.1107/S2056989019006935

**Published:** 2019-05-24

**Authors:** Lars Eriksson, Göran Widmalm

**Affiliations:** a Department of Materials and Environmental Chemistry, Stockholm University, SE-106 91, Stockholm, Sweden; b Department of Organic Chemistry, Stockholm University, SE-106 91, Stockholm, Sweden

**Keywords:** crystal structure, carbohydrates, disaccharide, conformation, packing

## Abstract

The title compound, C_13_H_24_O_9_·H_2_O, a structural model for part of bacterial O-anti­gen polysaccharides from *Shigella flexneri* and *Escherichia coli*, crystallizes with four independent disaccharide mol­ecules and four water mol­ecules in the asymmetric unit. The conformation at the glycosidic linkage joining the two rhamnosyl residues is described by the torsion angles φ_H_ of 39, 30, 37 and 37°, and ψ_H_ of −32, −35, −31 and −32°, which are the major conformation region known to be populated in an aqueous solution. The hexo­pyran­ose rings have the ^1^ *C*_4_ chair conformation. In the crystal, the disaccharide and water mol­ecules are associated through O—H⋯O hydrogen bonds, forming a layer parallel to the *bc* plane. The layers stack along the *a* axis *via* hydro­phobic inter­actions between the methyl groups.

## Abstract 2

The title compound crystallizes with four unique disaccharide mol­ecules and four water mol­ecules in the asymmetric unit. In the crystal, the disaccharide and water mol­ecules form layers parallel to the *bc* plane *via* hydro­philic inter­actions.

## Chemical context   

The title disaccharide compound is a structural model for part of bacterial O-anti­gen polysaccharides from *Shigella flexneri* (Kubler-Kielb *et al.*, 2007 ▸) and *Escherichia coli* (Marie *et al.*, 1998 ▸). In the title compound, inter-residue hydrogen bonding is not possible, which thus gives the opportunity to study conformational preferences at the glycosidic linkage devoid of the hydrogen bonds. Furthermore, the major conformation in water differs from that in dimethyl sulfoxide as determined by NMR spectroscopy and mol­ecular dynamics simulations (Pendrill *et al.*, 2016 ▸). These conformations can be compared to the present crystal structure obtained from a water:ethanol (1:1) mixed solution.
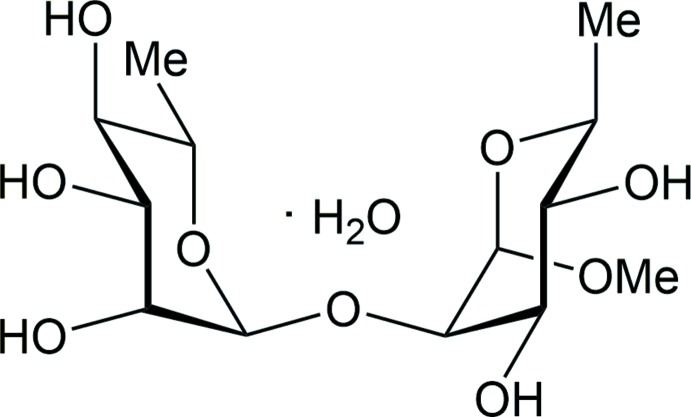


## Structural commentary   

**Figure 1:**
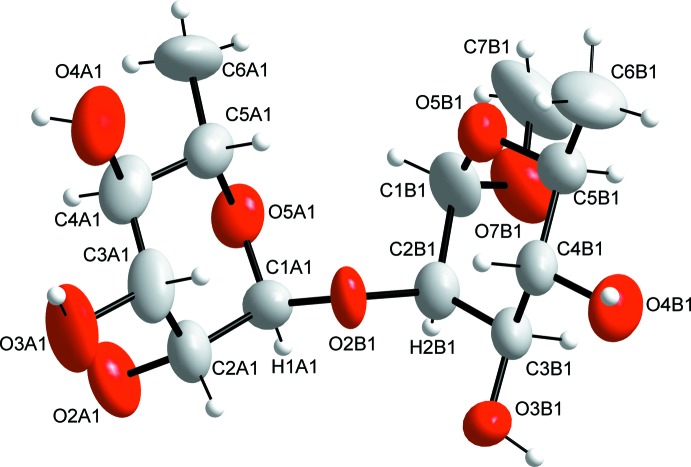
The structure of one of the title disaccharide mol­ecules, disaccharide 1, showing the atom-labelling scheme. The third character of the atom label denotes the rhamnose residue A or B in each disaccharide and the fourth character indicates each independent disaccharide entity. Displacement ellipsoids are drawn at the 50% probability level.

**Figure 2:**
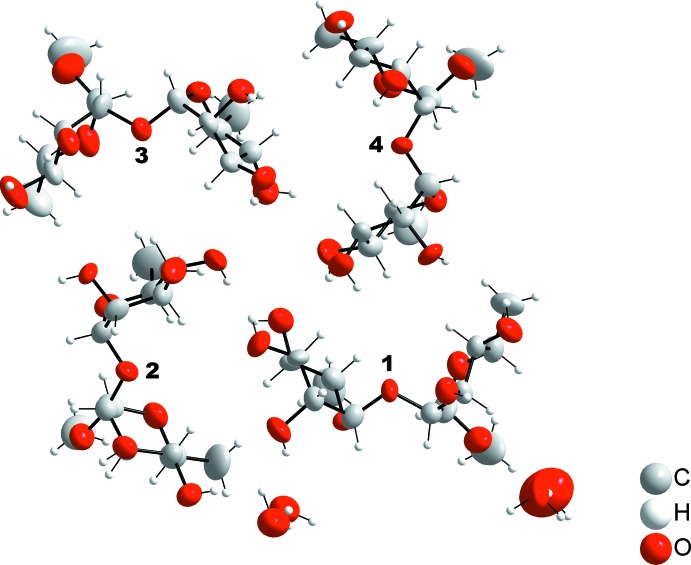
The four independent disaccharide mol­ecules, 1–4, in the asymmetric unit together with four adjacent water mol­ecules.

The asymmetric unit of the title compound contains four independent disaccharides of closely similar conformation, shown in Figs. 1 ▸–3 ▸ ▸, where the hexo­pyran­ose rings have the ^1^ *C*_4_ chair conformation. In the disaccharide mol­ecule, there are three major degrees of freedom with the glycosidic torsion angles of φ_H_, ψ_H_ and φ_H_(C7), which are defined, respectively, by H1*A*—C1*A*—O2*B*—C2*B*, C1*A*—O2*B*—C2*B*—H2*B* and H1*B*—C1*B*—O7*B*—C7*B*. These torsion angles are (I) φ_H_ =39°, ψ_H_ = −32° and φ_H_(C7) = 49°, (II) φ_H_ = 30°, ψ_H_ −35° and φ_H_(C7) = 52°, (III) φ_H_ = 36°, ψ_H_ = −31° and φ_H_(C7) = 51°, and (IV) φ_H_ = 37°, ψ_H_ = −32° and φ_H_(C7) = 51°, where (I)–(IV) correspond to the four independent disaccharide mol­ecules 1–4, respectively, in Fig. 2 ▸. The average φ_H_, ψ_H_ and φ_H_(C7) angles are 35 (4), −33 (2) and 51 (1)°, respectively. The φ_H_ torsion angle is governed by the exo-anomeric effect and should be approximately 40° for an α-l-sugar, which is also the case in the title rhamnose-containing disaccharide (Widmalm *et al.*, 1992 ▸). The ψ_H_ torsion angle depends on the stereochemistry at or close to the glycosidic linkage. In solution it can take both positive and negative values, depending on the solvent that the solute is dissolved in (Pendrill *et al.*, 2016 ▸). Inter­estingly, in the crystal of the title compound, the ψ_H_ torsion angle is negative like the major conformer in water solution (Pendrill *et al.*, 2016 ▸). This conformation causes the three methyl groups to be positioned on one side of the mol­ecule. In the crystal of a rhamnose-containing tris­accharide having the glycosidic α-(1 → 2)-linkage (Eriksson & Widmalm, 2012 ▸), quite similar torsion angles of φ_H_ = 48° and ψ_H_ = −29° were observed.

## Supra­molecular features   

Hydro­philic inter­actions dominate in a network of O—H⋯O hydrogen bonds that connect the disaccharide and water mol­ecules (Table 1 ▸), forming a layer parallel to the *bc* plane, while hydro­phobic inter­actions between the methyl groups dominate in the *bc* plane at *x* = 0.5 (Figs. 2 ▸ and 4 ▸). A DFT optimization of the title structure has been performed with plane waves and pseudo potentials using *NWChem* (Valiev *et al.*, 2010 ▸). The major differences between the optimized and observed structures are that the O—H distances are slightly longer in the optimized structure than the experimental values and some geometrical details, *e.g.* torsion angles of hydroxyl groups. The hydrogen-bonding scheme obtained from the DFT-optimized structure was similar, with minor differences between the experimental structure and the DFT-optimized version.

## Database survey   

A search for related compounds in the CSD (2019 release; Groom *et al.*, 2016 ▸) gave only one hit with the rhamnose dimer as fragment, XEBQAY (Eriksson & Widmalm, 2012 ▸), with a good fit to the three-dimensional arrangement of the disaccharide element. A search using only the monomer skeleton without hydroxyl H atoms produced 178 hits, but most of these were not relevant for comparison with the title mol­ecule.

## Synthesis and crystallization   

The title compound was synthesized according to the published procedures (Norberg *et al.*, 1986 ▸), where the rhamnosyl residues have the *L* absolute configuration. Colourless prismatic single crystals were obtained by slow evaporation from a water:ethanol (1:1) mixture solution at ambient temperature.

## Refinement   

Crystal data, data collection and structural refinement details are summarized in Table 2 ▸. Diffraction data from three separate crystals of the approximately same size were merged using the *BASF* instruction available in the *SHELXL* program. Although each single crystal showed considerable disorder, the three crystals together provided a complete data set at the expense of a rather high inter­nal *R* value. Weak *ISOR* restraints were applied for all non-H atoms. H atoms in the disaccharide mol­ecules were added geometrically (C—H = 1.00 or 0.98 Å and O—H = 0.84 Å) and treated as riding with *U*_iso_(H) = 1.2–1.5*U*_eq_(C,O). The O—H bond and H⋯H distances in the water mol­ecules were restrained to 0.85 (1) and 1.34 (1) Å, respectively. The orientation of each water mol­ecule was adjusted and restrained with additional *DFIX* commands using parameters derived from a solid state DFT optimization of the crystal structure.

## Supplementary Material

Crystal structure: contains datablock(s) I, global. DOI: 10.1107/S2056989019006935/is5512sup1.cif

Structure factors: contains datablock(s) I. DOI: 10.1107/S2056989019006935/is5512Isup2.hkl

CCDC reference: 1915954

Additional supporting information:  crystallographic information; 3D view; checkCIF report

## Figures and Tables

**Figure 3:**
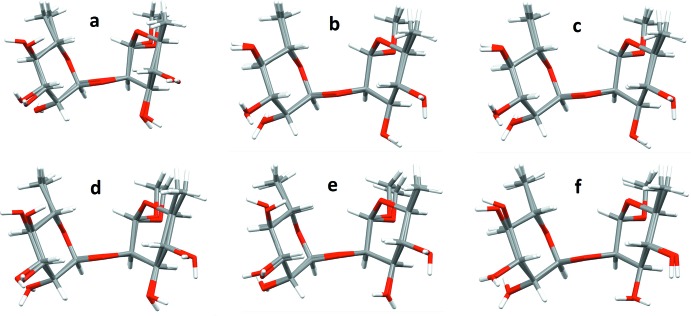
Overlays between pairs of the four independent mol­ecules with minimal root-mean-square deviations (RMSD): (*a*) 1 and 2, (*b*) 1 and 3, (*c*) 1 and 4, (*d*) 2 and 3, (*e*) 2 and 4, (*f*) 3 and 4.

**Figure 4:**
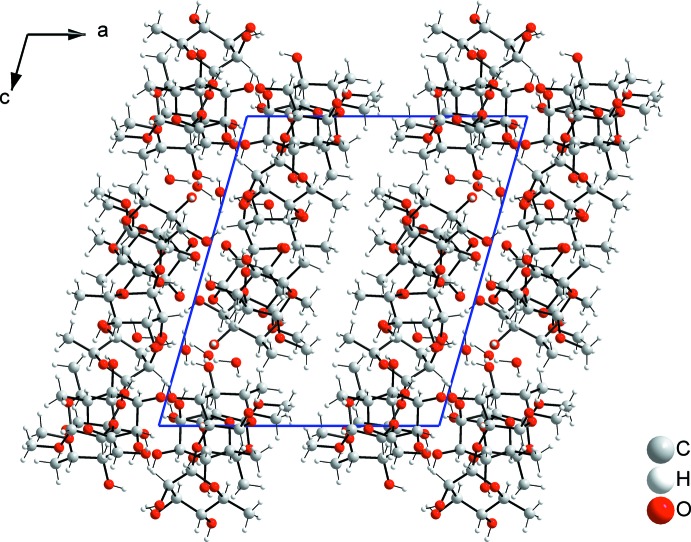
A packing diagram of the title compound viewed along the *b* axis, showing hydro­philic and hydro­phobic contacts between layers. The hydro­philic bound layers extend parallel to the *bc* plane, while the layers pack with hydro­phobic inter­actions at *x* = 0.5.

**Table 1:** Hydrogen-bond geometry (Å, °)

*D*—H⋯*A*	*D*—H	H⋯*A*	*D*⋯*A*	*D*—H⋯*A*
O2*A*1—H2*A*2⋯O2	0.84	1.96	2.770 (9)	161
O3*A*1—H3*A*2⋯O4*A*2	0.84	2.59	3.366 (10)	154
O3*B*1—H3*B*2⋯O3*A*3^i^	0.84	1.95	2.766 (8)	164
O4*B*1—H4*B*2⋯O1^ii^	0.84	1.89	2.693 (10)	158
O2*A*2—H2*A*4⋯O1^iii^	0.84	2.04	2.834 (10)	159
O3*A*2—H3*A*4⋯O3^ii^	0.84	1.85	2.647 (9)	159
O4*A*2—H4*A*4⋯O4*A*1	0.84	2.09	2.862 (8)	152
O3*B*2—H3*B*4⋯O3*A*2^iv^	0.84	2.06	2.710 (7)	133
O4*B*2—H4*B*4⋯O2	0.84	2.20	2.952 (8)	150
O2*A*3—H2*A*6⋯O4*B*1^ii^	0.84	1.98	2.791 (8)	161
O3*A*3—H3*A*6⋯O3^ii^	0.84	1.92	2.741 (9)	165
O4*A*3—H4*A*6⋯O4*A*4	0.84	2.13	2.731 (9)	128
O3*B*3—H3*B*6⋯O2*A*1^iii^	0.84	2.58	3.309 (8)	146
O3*B*3—H3*B*6⋯O3*A*1^iii^	0.84	2.13	2.855 (8)	145
O4*B*3—H4*B*6⋯O2*A*1^iii^	0.84	2.00	2.754 (9)	149
C3*A*4—H3*A*7⋯O3*A*3	1.00	2.56	3.431 (10)	146
O3*A*4—H3*A*8⋯O3*B*4^i^	0.84	2.08	2.761 (8)	138
O4*A*4—H4*A*8⋯O4*A*1	0.84	2.05	2.717 (9)	136
O3*B*4—H3*B*8⋯O3*B*1^ii^	0.84	2.02	2.843 (7)	168
O4*B*4—H4*B*8⋯O2*A*4^ii^	0.84	2.08	2.859 (8)	155
O1—H12⋯O4*B*3^iv^	0.86 (1)	1.86 (2)	2.718 (10)	174 (7)
O2—H21⋯O4*B*4^v^	0.85 (1)	2.29 (4)	3.030 (9)	145 (6)
O2—H22⋯O5*A*3^v^	0.85 (1)	2.62 (2)	3.461 (9)	169 (5)
O3—H31⋯O3*A*4^i^	0.85 (1)	2.00 (4)	2.695 (8)	139 (5)
O3—H32⋯O4	0.85 (1)	1.80 (3)	2.582 (13)	152 (7)
O4—H41⋯O7*B*1	0.85 (1)	2.29 (3)	3.037 (14)	147 (5)
O4—H42⋯O7*B*3^vi^	0.85 (1)	2.26 (3)	3.021 (14)	148 (4)

Symmetry codes: (i) 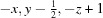; (ii) 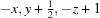; (iii) 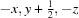; (iv) 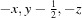; (v) 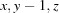; (vi) 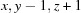.

**Table 2:** Experimental details

Crystal data	
Chemical formula	C_13_H_24_O_9_·H_2_O
*M*_r_	342.34
Crystal system, space group	Monoclinic, *P*2_1_
Temperature (K)	100
*a*, *b*, *c* (Å)	13.936 (3), 15.501 (3), 15.988 (3)
β (°)	105.92 (16)
*V* (Å^3^)	3321 (12)
*Z*	8
Radiation type	Cu *K*α
μ (mm^−1^)	1.02
Crystal size (mm)	0.10 × 0.07 × 0.03

Data collection	
Diffractometer	Bruker D8 Advance
Absorption correction	Multi-scan (*APEX3*; Bruker, 2017 ▸)
*T*_min_, *T*_max_	0.90, 0.97
No. of measured, independent and observed [*I* > 2σ(*I*)] reflections	42501, 11996, 4360
*R*_int_	0.158
(sin θ/λ)_max_ (Å^−1^)	0.602

Refinement	
*R*[*F* ^2^ > 2σ(*F* ^2^)], *wR*(*F* ^2^), *S*	0.075, 0.173, 0.85
No. of reflections	11996
No. of parameters	885
No. of restraints	571
H-atom treatment	H atoms treated by a mixture of independent and constrained refinement
Δρ_max_, Δρ_min_ (e Å^−3^)	0.44, −0.30
Absolute structure	Flack *x* determined using 1400 quotients [(*I* ^+^)−(*I* ^−^)]/[(*I* ^+^)+(*I* ^−^)] (Parsons *et al.*, 2013 ▸)
Absolute structure parameter	−0.03 (18)

Computer programs: *APEX3* (Bruker, 2017 ▸), *CrysAlis PRO* (Agilent, 2014 ▸), *SHELXT* (Sheldrick, 2015*a* ▸), *SHELXL2016* (Sheldrick, 2015*b* ▸), *DIAMOND* (Brandenburg, 1999 ▸), *Mercury* (Macrae *et al.*, 2008 ▸), *PLATON* (Spek, 2009 ▸), *enCIFer* (Allen *et al.*, 2004 ▸) and *publCIF* (Westrip, 2010 ▸).

## supplementary crystallographic information

### Crystal data

**Table:**

C_13_H_24_O_9_·H_2_O	*F*(000) = 1472
*M**_r_* = 342.34	*D*_x_ = 1.369 Mg m^−^^3^
Monoclinic, *P*2_1_	Cu *K*α radiation, λ = 1.54184 Å
*a* = 13.936 (3) Å	Cell parameters from 29997 reflections
*b* = 15.501 (3) Å	θ = 2.8–68.2°
*c* = 15.988 (3) Å	µ = 1.02 mm^−^^1^
β = 105.92 (16)°	*T* = 100 K
*V* = 3321 (12) Å^3^	Prism, colourless
*Z* = 8	0.10 × 0.07 × 0.03 mm

### Data collection

**Table:**

Bruker D8 Advance diffractometer	11996 independent reflections
Radiation source: Incotec 1myS	4360 reflections with *I* > 2σ(*I*)
Detector resolution: 10 pixels mm^-1^	*R*_int_ = 0.158
ω scans at different φ	θ_max_ = 68.3°, θ_min_ = 2.9°
Absorption correction: multi-scan (APEX3; Bruker, 2017)	*h* = −16→16
*T*_min_ = 0.90, *T*_max_ = 0.97	*k* = −18→18
42501 measured reflections	*l* = −19→12

### Refinement

**Table:**

Refinement on *F*^2^	Hydrogen site location: mixed
Least-squares matrix: full	H atoms treated by a mixture of independent and constrained refinement
*R*[*F*^2^ > 2σ(*F*^2^)] = 0.075	*w* = 1/[σ^2^(*F*_o_^2^) + (0.0773*P*)^2^] where *P* = (*F*_o_^2^ + 2*F*_c_^2^)/3
*wR*(*F*^2^) = 0.173	(Δ/σ)_max_ < 0.001
*S* = 0.85	Δρ_max_ = 0.44 e Å^−^^3^
11996 reflections	Δρ_min_ = −0.30 e Å^−^^3^
885 parameters	Absolute structure: Flack *x* determined using 1400 quotients [(*I*^+^)-(*I*^-^)]/[(*I*^+^)+(*I*^-^)] (Parsons *et al*., 2013)
571 restraints	Absolute structure parameter: −0.03 (18)

### Special details

**Table:**

Geometry. All esds (except the esd in the dihedral angle between two l.s. planes) are estimated using the full covariance matrix. The cell esds are taken into account individually in the estimation of esds in distances, angles and torsion angles; correlations between esds in cell parameters are only used when they are defined by crystal symmetry. An approximate (isotropic) treatment of cell esds is used for estimating esds involving l.s. planes.

### Fractional atomic coordinates and isotropic or equivalent isotropic displacement parameters (Å^2^)

**Table:**

	*x*	*y*	*z*	*U*_iso_*/*U*_eq_	
C1A1	0.1588 (5)	0.4641 (5)	0.4294 (5)	0.055 (2)	
H1A1	0.127984	0.406281	0.432176	0.067*	
C2A1	0.0970 (6)	0.5092 (5)	0.3498 (5)	0.064 (2)	
H2A1	0.030768	0.524470	0.358759	0.076*	
C3A1	0.1466 (7)	0.5887 (5)	0.3330 (5)	0.067 (3)	
H3A1	0.146376	0.630796	0.380328	0.080*	
C4A1	0.2544 (7)	0.5734 (5)	0.3331 (5)	0.069 (3)	
H4A1	0.255387	0.534858	0.283178	0.083*	
C5A1	0.3088 (6)	0.5291 (6)	0.4169 (5)	0.065 (2)	
H5A1	0.308327	0.567829	0.466795	0.078*	
C6A1	0.4145 (5)	0.5059 (7)	0.4220 (6)	0.096 (3)	
H6A1	0.439348	0.464391	0.469170	0.144*	
H6A2	0.455910	0.558038	0.433340	0.144*	
H6A3	0.417529	0.480247	0.366752	0.144*	
O2A1	0.0818 (5)	0.4538 (4)	0.2760 (3)	0.0843 (19)	
H2A2	0.111548	0.406835	0.291124	0.126*	
O3A1	0.0921 (5)	0.6257 (4)	0.2525 (3)	0.095 (2)	
H3A2	0.113673	0.675525	0.247976	0.142*	
O4A1	0.3012 (5)	0.6531 (4)	0.3232 (4)	0.094 (2)	
H4A2	0.361327	0.651805	0.352438	0.141*	
O5A1	0.2583 (4)	0.4503 (3)	0.4257 (3)	0.0609 (15)	
C1B1	0.2893 (6)	0.4581 (6)	0.6248 (5)	0.074 (3)	
H1B1	0.323667	0.444539	0.579123	0.089*	
C2B1	0.1752 (6)	0.4588 (5)	0.5821 (5)	0.054 (2)	
H2B1	0.150782	0.398510	0.567459	0.064*	
C3B1	0.1192 (5)	0.4997 (5)	0.6395 (5)	0.052 (2)	
H3B1	0.122395	0.459261	0.688919	0.062*	
C4B1	0.1666 (6)	0.5834 (5)	0.6776 (5)	0.054 (2)	
H4B1	0.161154	0.626339	0.629919	0.065*	
C5B1	0.2773 (6)	0.5682 (6)	0.7247 (5)	0.060 (2)	
H5B1	0.282756	0.523772	0.771080	0.072*	
C6B1	0.3288 (6)	0.6512 (6)	0.7658 (6)	0.089 (3)	
H6B1	0.401346	0.643363	0.781227	0.133*	
H6B2	0.308315	0.664662	0.818240	0.133*	
H6B3	0.309780	0.698809	0.724139	0.133*	
C7B1	0.4048 (8)	0.3660 (7)	0.7137 (6)	0.127 (4)	
H7B1	0.425329	0.338345	0.666205	0.191*	
H7B2	0.413196	0.325296	0.762072	0.191*	
H7B3	0.446203	0.417110	0.733497	0.191*	
O7B1	0.3057 (5)	0.3903 (4)	0.6845 (4)	0.093 (2)	
O2B1	0.1544 (3)	0.5105 (3)	0.5036 (3)	0.0533 (14)	
O3B1	0.0180 (3)	0.5117 (4)	0.5954 (3)	0.0612 (16)	
H3B2	−0.016989	0.503039	0.629779	0.092*	
O4B1	0.1147 (4)	0.6154 (4)	0.7377 (4)	0.0773 (17)	
H4B2	0.112170	0.669516	0.734996	0.116*	
O5B1	0.3246 (4)	0.5372 (4)	0.6627 (3)	0.0715 (17)	
C1A2	0.1581 (6)	0.6656 (5)	−0.0379 (5)	0.054 (2)	
H1A2	0.141765	0.655735	−0.102051	0.065*	
C2A2	0.0717 (5)	0.7139 (5)	−0.0176 (5)	0.053 (2)	
H2A3	0.011101	0.676328	−0.032092	0.064*	
C3A2	0.0991 (6)	0.7355 (5)	0.0768 (5)	0.056 (2)	
H3A3	0.101335	0.680392	0.109826	0.067*	
C4A2	0.2001 (6)	0.7773 (5)	0.1074 (5)	0.056 (2)	
H4A3	0.195294	0.836153	0.081066	0.067*	
C5A2	0.2795 (6)	0.7281 (5)	0.0799 (5)	0.063 (3)	
H5A2	0.292157	0.672268	0.112451	0.075*	
C6A2	0.3764 (6)	0.7779 (7)	0.0972 (7)	0.108 (4)	
H6A4	0.420797	0.749581	0.067584	0.162*	
H6A5	0.408552	0.779393	0.159934	0.162*	
H6A6	0.362618	0.836938	0.075304	0.162*	
O2A2	0.0514 (4)	0.7907 (3)	−0.0689 (3)	0.0613 (15)	
H2A4	0.054395	0.779870	−0.119609	0.092*	
O3A2	0.0224 (4)	0.7870 (4)	0.0924 (4)	0.0822 (18)	
H3A4	0.018315	0.778083	0.143086	0.123*	
O4A2	0.2259 (5)	0.7869 (4)	0.1991 (3)	0.099 (2)	
H4A4	0.259062	0.743856	0.222744	0.148*	
O5A2	0.2486 (4)	0.7104 (3)	−0.0111 (3)	0.0638 (16)	
C1B2	0.3082 (5)	0.5083 (5)	−0.0125 (5)	0.060 (2)	
H1B2	0.335341	0.566739	−0.019539	0.072*	
C2B2	0.1929 (5)	0.5118 (5)	−0.0371 (5)	0.055 (2)	
H2B2	0.166629	0.518370	−0.101526	0.066*	
C3B2	0.1505 (5)	0.4336 (5)	−0.0098 (4)	0.051 (2)	
H3B3	0.163201	0.384344	−0.045701	0.061*	
C4B2	0.1956 (5)	0.4125 (5)	0.0827 (5)	0.050 (2)	
H4B3	0.182263	0.459998	0.120421	0.060*	
C5B2	0.3071 (6)	0.4009 (6)	0.0978 (5)	0.070 (3)	
H5B2	0.318640	0.354420	0.058308	0.084*	
C6B2	0.3625 (7)	0.3789 (7)	0.1885 (5)	0.101 (4)	
H6B4	0.433846	0.389629	0.197108	0.152*	
H6B5	0.352109	0.317818	0.199425	0.152*	
H6B6	0.338144	0.414573	0.228987	0.152*	
C7B2	0.4356 (6)	0.4518 (7)	−0.0634 (6)	0.100 (3)	
H7B4	0.456184	0.511146	−0.070420	0.150*	
H7B5	0.449567	0.415255	−0.108681	0.150*	
H7B6	0.472685	0.430111	−0.006015	0.150*	
O7B2	0.3331 (4)	0.4500 (4)	−0.0705 (3)	0.0729 (17)	
O2B2	0.1629 (4)	0.5847 (3)	0.0046 (3)	0.0569 (14)	
O3B2	0.0439 (4)	0.4440 (3)	−0.0269 (3)	0.0600 (15)	
H3B4	0.014909	0.415542	−0.071342	0.090*	
O4B2	0.1553 (4)	0.3332 (3)	0.1036 (3)	0.0721 (17)	
H4B4	0.143650	0.337695	0.152231	0.108*	
O5B2	0.3466 (4)	0.4799 (4)	0.0755 (3)	0.0722 (17)	
C1A3	0.1619 (5)	1.1095 (5)	0.1451 (5)	0.051 (2)	
H1A3	0.135321	1.168477	0.126627	0.061*	
C2A3	0.0957 (6)	1.0671 (5)	0.1914 (4)	0.052 (2)	
H2A5	0.029195	1.056076	0.149195	0.062*	
C3A3	0.1369 (5)	0.9837 (5)	0.2338 (5)	0.052 (2)	
H3A5	0.135203	0.940673	0.186805	0.063*	
C4A3	0.2418 (5)	0.9938 (5)	0.2858 (5)	0.054 (2)	
H4A5	0.242986	1.029522	0.338150	0.065*	
C5A3	0.3051 (6)	1.0385 (6)	0.2349 (5)	0.066 (2)	
H5A3	0.307544	0.999564	0.185690	0.079*	
C6A3	0.4115 (6)	1.0609 (7)	0.2829 (6)	0.106 (4)	
H6A7	0.438733	1.100650	0.247490	0.159*	
H6A8	0.451856	1.008213	0.293832	0.159*	
H6A9	0.413012	1.088516	0.338372	0.159*	
O2A3	0.0834 (4)	1.1262 (3)	0.2569 (3)	0.0685 (16)	
H2A6	0.029325	1.115795	0.268637	0.103*	
O3A3	0.0777 (5)	0.9516 (4)	0.2858 (3)	0.0782 (18)	
H3A6	0.050509	0.905243	0.264422	0.117*	
O4A3	0.2877 (5)	0.9112 (4)	0.3149 (4)	0.085 (2)	
H4A6	0.286590	0.902422	0.366463	0.128*	
O5A3	0.2605 (4)	1.1172 (3)	0.1981 (3)	0.0632 (15)	
C1B3	0.2909 (6)	1.1088 (7)	0.0104 (6)	0.073 (3)	
H1B3	0.325651	1.121000	0.072772	0.088*	
C2B3	0.1787 (5)	1.1089 (6)	−0.0019 (4)	0.056 (2)	
H2B3	0.155292	1.169631	−0.000128	0.067*	
C3B3	0.1196 (6)	1.0669 (5)	−0.0853 (4)	0.052 (2)	
H3B5	0.120980	1.108243	−0.133006	0.062*	
C4B3	0.1680 (6)	0.9869 (6)	−0.1041 (4)	0.062 (2)	
H4B5	0.164335	0.941940	−0.060292	0.075*	
C5B3	0.2751 (6)	1.0024 (7)	−0.1007 (5)	0.074 (3)	
H5B3	0.278136	1.049188	−0.142897	0.089*	
C6B3	0.3292 (7)	0.9238 (6)	−0.1216 (6)	0.101 (4)	
H6B7	0.399856	0.937750	−0.113055	0.152*	
H6B8	0.299487	0.906787	−0.182222	0.152*	
H6B9	0.323294	0.876152	−0.083050	0.152*	
C7B3	0.4102 (8)	1.1990 (8)	−0.0228 (8)	0.148 (5)	
H7B7	0.435520	1.214803	0.038717	0.222*	
H7B8	0.419358	1.247511	−0.059113	0.222*	
H7B9	0.446993	1.148735	−0.034851	0.222*	
O7B3	0.3070 (5)	1.1785 (4)	−0.0417 (4)	0.105 (2)	
O2B3	0.1578 (3)	1.0598 (3)	0.0698 (3)	0.0567 (15)	
O3B3	0.0181 (4)	1.0544 (4)	−0.0898 (3)	0.0655 (16)	
H3B6	−0.015465	1.052127	−0.142175	0.098*	
O4B3	0.1184 (4)	0.9558 (4)	−0.1893 (3)	0.089 (2)	
H4B6	0.057905	0.969962	−0.202061	0.133*	
O5B3	0.3258 (4)	1.0321 (4)	−0.0136 (3)	0.0769 (18)	
C1A4	0.1636 (6)	0.9094 (5)	0.6101 (5)	0.058 (2)	
H1A4	0.140914	0.920053	0.663321	0.070*	
C2A4	0.0871 (6)	0.8537 (5)	0.5488 (5)	0.052 (2)	
H2A7	0.024405	0.888022	0.526967	0.062*	
C3A4	0.1254 (7)	0.8283 (5)	0.4709 (5)	0.064 (3)	
H3A7	0.127031	0.881835	0.436413	0.076*	
C4A4	0.2312 (6)	0.7923 (6)	0.4990 (5)	0.066 (2)	
H4A7	0.230144	0.734660	0.526807	0.079*	
C5A4	0.2970 (6)	0.8528 (5)	0.5643 (5)	0.065 (3)	
H5A4	0.304319	0.908045	0.534381	0.078*	
C6A4	0.4013 (6)	0.8124 (7)	0.6014 (6)	0.101 (4)	
H6AX	0.395287	0.759364	0.632966	0.151*	
H6AY	0.444527	0.853353	0.641254	0.151*	
H6AZ	0.430358	0.798874	0.553676	0.151*	
O2A4	0.0650 (4)	0.7793 (3)	0.5922 (3)	0.0698 (16)	
H2A8	0.118336	0.757990	0.623257	0.105*	
O3A4	0.0571 (5)	0.7715 (4)	0.4175 (3)	0.0838 (19)	
H3A8	0.058597	0.723941	0.442986	0.126*	
O4A4	0.2691 (5)	0.7829 (4)	0.4265 (4)	0.104 (2)	
H4A8	0.283653	0.730958	0.421495	0.157*	
O5A4	0.2571 (4)	0.8711 (3)	0.6351 (3)	0.0576 (15)	
C1B4	0.3105 (6)	1.0699 (6)	0.6460 (5)	0.066 (3)	
H1B4	0.339548	1.011305	0.662436	0.079*	
C2B4	0.1981 (6)	1.0627 (5)	0.6225 (5)	0.052 (2)	
H2B4	0.176932	1.054685	0.676962	0.062*	
C3B4	0.1467 (6)	1.1400 (5)	0.5753 (5)	0.052 (2)	
H3B7	0.159154	1.188506	0.618196	0.062*	
C4B4	0.1886 (6)	1.1666 (5)	0.5045 (5)	0.055 (2)	
H4B7	0.176363	1.119696	0.459838	0.066*	
C5B4	0.2989 (6)	1.1816 (5)	0.5376 (6)	0.063 (3)	
H5B4	0.311840	1.225543	0.585160	0.076*	
C6B4	0.3527 (8)	1.2078 (7)	0.4718 (6)	0.104 (4)	
H6BX	0.424430	1.197486	0.495791	0.156*	
H6BY	0.341100	1.269160	0.458211	0.156*	
H6BZ	0.327713	1.173650	0.418693	0.156*	
C7B4	0.4500 (7)	1.1195 (7)	0.7541 (6)	0.133 (5)	
H7BX	0.471074	1.059044	0.761509	0.199*	
H7BY	0.468386	1.148560	0.810687	0.199*	
H7BZ	0.483055	1.148171	0.714860	0.199*	
O7B4	0.3427 (5)	1.1237 (4)	0.7172 (4)	0.091 (2)	
O2B4	0.1664 (4)	0.9902 (3)	0.5675 (3)	0.0579 (15)	
O3B4	0.0422 (4)	1.1278 (3)	0.5471 (3)	0.0642 (15)	
H3B8	0.028754	1.088056	0.510029	0.096*	
O4B4	0.1443 (4)	1.2441 (3)	0.4640 (4)	0.0746 (17)	
H4B8	0.082866	1.243590	0.459889	0.112*	
O5B4	0.3421 (4)	1.0997 (4)	0.5733 (4)	0.0736 (17)	
O1	−0.1048 (6)	0.2846 (5)	0.2275 (5)	0.147 (3)	
H11	−0.0401 (12)	0.279 (3)	0.245 (7)	0.177*	
H12	−0.111 (3)	0.3377 (16)	0.211 (5)	0.177*	
O2	0.1643 (6)	0.2903 (4)	0.2855 (4)	0.116 (2)	
H21	0.186 (5)	0.277 (4)	0.3388 (10)	0.139*	
H22	0.190 (4)	0.253 (3)	0.259 (2)	0.139*	
O3	0.0171 (6)	0.2961 (5)	0.7551 (4)	0.131 (3)	
H31	−0.005 (4)	0.263 (3)	0.712 (2)	0.157*	
H32	0.076 (2)	0.278 (4)	0.779 (3)	0.157*	
O4	0.2092 (8)	0.2831 (10)	0.7982 (8)	0.249 (6)	
H41	0.234 (3)	0.293 (4)	0.7562 (19)	0.299*	
H42	0.246 (4)	0.243 (2)	0.828 (2)	0.299*	

### Atomic displacement parameters (Å^2^)

**Table:**

	*U*^11^	*U*^22^	*U*^33^	*U*^12^	*U*^13^	*U*^23^
C1A1	0.050 (5)	0.059 (6)	0.058 (5)	0.001 (5)	0.017 (4)	0.009 (5)
C2A1	0.088 (6)	0.061 (6)	0.042 (5)	0.005 (5)	0.018 (5)	0.000 (5)
C3A1	0.117 (8)	0.030 (5)	0.058 (5)	−0.002 (5)	0.032 (5)	0.000 (4)
C4A1	0.116 (7)	0.034 (6)	0.066 (6)	−0.015 (5)	0.039 (5)	−0.004 (5)
C5A1	0.080 (6)	0.063 (6)	0.058 (5)	−0.012 (5)	0.028 (5)	−0.011 (5)
C6A1	0.044 (6)	0.142 (10)	0.102 (7)	−0.007 (6)	0.018 (5)	−0.018 (7)
O2A1	0.124 (5)	0.068 (4)	0.052 (3)	0.008 (4)	0.010 (3)	−0.020 (3)
O3A1	0.159 (6)	0.063 (4)	0.057 (4)	0.017 (4)	0.020 (4)	0.026 (3)
O4A1	0.134 (6)	0.072 (5)	0.079 (4)	−0.029 (4)	0.036 (4)	−0.011 (4)
O5A1	0.088 (4)	0.039 (3)	0.060 (3)	0.005 (3)	0.027 (3)	0.004 (3)
C1B1	0.076 (7)	0.080 (7)	0.059 (6)	0.034 (6)	0.007 (5)	0.020 (6)
C2B1	0.065 (6)	0.047 (5)	0.049 (5)	0.001 (4)	0.015 (4)	0.001 (4)
C3B1	0.057 (5)	0.060 (6)	0.037 (4)	−0.010 (5)	0.010 (4)	−0.005 (4)
C4B1	0.072 (6)	0.052 (6)	0.040 (4)	−0.010 (5)	0.018 (4)	0.000 (4)
C5B1	0.063 (6)	0.070 (6)	0.045 (5)	−0.010 (5)	0.012 (4)	0.003 (5)
C6B1	0.077 (7)	0.063 (7)	0.110 (7)	−0.016 (5)	−0.001 (6)	−0.022 (6)
C7B1	0.124 (10)	0.112 (10)	0.104 (8)	0.040 (8)	−0.041 (7)	0.002 (7)
O7B1	0.118 (6)	0.069 (4)	0.075 (4)	0.016 (4)	−0.002 (4)	0.013 (4)
O2B1	0.075 (3)	0.055 (4)	0.030 (3)	0.011 (3)	0.014 (3)	0.003 (3)
O3B1	0.053 (3)	0.080 (4)	0.053 (3)	−0.015 (3)	0.018 (3)	−0.002 (3)
O4B1	0.096 (4)	0.071 (4)	0.073 (4)	−0.006 (4)	0.036 (3)	−0.021 (3)
O5B1	0.074 (4)	0.084 (5)	0.059 (3)	−0.003 (3)	0.023 (3)	−0.004 (3)
C1A2	0.067 (6)	0.050 (6)	0.045 (5)	−0.012 (5)	0.015 (4)	0.009 (4)
C2A2	0.042 (5)	0.050 (6)	0.070 (6)	−0.010 (4)	0.020 (4)	0.009 (5)
C3A2	0.077 (6)	0.054 (6)	0.043 (5)	0.006 (5)	0.025 (5)	0.004 (4)
C4A2	0.086 (6)	0.037 (5)	0.044 (5)	0.008 (5)	0.017 (5)	0.003 (4)
C5A2	0.066 (6)	0.056 (6)	0.056 (6)	−0.005 (5)	0.000 (5)	0.010 (5)
C6A2	0.052 (6)	0.117 (8)	0.140 (9)	−0.019 (6)	0.000 (6)	−0.003 (8)
O2A2	0.102 (4)	0.042 (3)	0.042 (3)	0.014 (3)	0.022 (3)	0.018 (3)
O3A2	0.115 (5)	0.067 (4)	0.080 (4)	0.027 (4)	0.055 (4)	0.011 (4)
O4A2	0.185 (7)	0.047 (4)	0.047 (4)	0.016 (4)	0.002 (4)	−0.008 (3)
O5A2	0.067 (4)	0.056 (4)	0.076 (4)	0.003 (3)	0.032 (3)	0.006 (3)
C1B2	0.062 (6)	0.057 (6)	0.064 (6)	0.010 (5)	0.021 (5)	−0.012 (5)
C2B2	0.058 (5)	0.059 (6)	0.050 (5)	0.005 (5)	0.017 (4)	0.001 (5)
C3B2	0.051 (6)	0.055 (6)	0.042 (5)	0.000 (4)	0.007 (4)	0.002 (4)
C4B2	0.055 (6)	0.048 (6)	0.044 (5)	−0.007 (4)	0.008 (4)	0.004 (4)
C5B2	0.070 (7)	0.065 (7)	0.071 (6)	0.000 (5)	0.011 (5)	0.004 (5)
C6B2	0.090 (8)	0.126 (9)	0.066 (6)	0.007 (7)	−0.014 (5)	0.020 (6)
C7B2	0.064 (6)	0.123 (9)	0.123 (8)	0.012 (6)	0.044 (6)	0.005 (7)
O7B2	0.087 (4)	0.072 (4)	0.065 (3)	0.012 (3)	0.030 (3)	−0.003 (3)
O2B2	0.079 (4)	0.043 (4)	0.055 (3)	0.004 (3)	0.027 (3)	−0.008 (3)
O3B2	0.057 (4)	0.055 (4)	0.060 (3)	−0.003 (3)	0.002 (3)	−0.010 (3)
O4B2	0.096 (4)	0.055 (4)	0.066 (4)	0.002 (3)	0.023 (3)	0.008 (3)
O5B2	0.078 (4)	0.073 (4)	0.056 (3)	−0.007 (3)	0.003 (3)	−0.011 (3)
C1A3	0.045 (5)	0.067 (6)	0.045 (5)	−0.015 (4)	0.022 (4)	−0.014 (4)
C2A3	0.067 (5)	0.052 (6)	0.038 (4)	0.001 (5)	0.017 (4)	−0.013 (4)
C3A3	0.067 (6)	0.044 (6)	0.053 (5)	0.005 (4)	0.028 (5)	−0.004 (4)
C4A3	0.057 (5)	0.051 (6)	0.052 (5)	0.009 (5)	0.012 (4)	0.003 (4)
C5A3	0.070 (6)	0.053 (6)	0.079 (6)	0.000 (5)	0.028 (5)	−0.014 (5)
C6A3	0.054 (6)	0.161 (11)	0.097 (7)	−0.009 (7)	0.011 (5)	−0.005 (7)
O2A3	0.074 (4)	0.062 (4)	0.078 (4)	0.001 (3)	0.036 (3)	−0.005 (3)
O3A3	0.121 (5)	0.059 (4)	0.068 (4)	−0.016 (4)	0.049 (3)	0.008 (3)
O4A3	0.113 (5)	0.073 (5)	0.066 (4)	0.021 (4)	0.018 (4)	0.001 (3)
O5A3	0.080 (4)	0.049 (4)	0.062 (3)	−0.010 (3)	0.023 (3)	−0.002 (3)
C1B3	0.062 (6)	0.094 (8)	0.075 (6)	−0.020 (6)	0.036 (5)	−0.009 (6)
C2B3	0.065 (6)	0.064 (6)	0.043 (5)	−0.002 (5)	0.023 (4)	0.014 (4)
C3B3	0.061 (6)	0.060 (6)	0.036 (4)	0.003 (5)	0.014 (4)	0.002 (4)
C4B3	0.080 (6)	0.068 (6)	0.040 (5)	0.019 (5)	0.018 (4)	−0.008 (5)
C5B3	0.068 (6)	0.110 (8)	0.049 (5)	0.013 (6)	0.025 (5)	0.009 (5)
C6B3	0.077 (7)	0.119 (9)	0.109 (7)	0.040 (6)	0.027 (6)	−0.031 (7)
C7B3	0.146 (10)	0.129 (11)	0.205 (13)	−0.053 (8)	0.111 (9)	−0.018 (9)
O7B3	0.117 (5)	0.099 (6)	0.125 (5)	−0.028 (4)	0.075 (4)	0.015 (4)
O2B3	0.073 (3)	0.057 (4)	0.047 (3)	−0.008 (3)	0.027 (3)	−0.005 (3)
O3B3	0.064 (4)	0.083 (4)	0.049 (3)	0.014 (3)	0.013 (3)	−0.002 (3)
O4B3	0.079 (4)	0.112 (5)	0.068 (4)	0.028 (4)	0.010 (3)	−0.032 (4)
O5B3	0.070 (4)	0.107 (5)	0.059 (4)	−0.003 (4)	0.027 (3)	−0.011 (4)
C1A4	0.046 (5)	0.064 (6)	0.063 (5)	−0.003 (5)	0.010 (4)	0.002 (5)
C2A4	0.061 (5)	0.039 (6)	0.052 (5)	0.015 (4)	0.010 (4)	0.006 (4)
C3A4	0.099 (7)	0.048 (6)	0.043 (5)	−0.022 (5)	0.019 (5)	−0.003 (4)
C4A4	0.086 (7)	0.052 (6)	0.067 (6)	−0.002 (5)	0.033 (5)	−0.003 (5)
C5A4	0.079 (6)	0.053 (6)	0.067 (6)	−0.018 (5)	0.023 (5)	−0.004 (5)
C6A4	0.051 (6)	0.133 (10)	0.121 (8)	0.032 (6)	0.027 (6)	0.018 (7)
O2A4	0.095 (4)	0.045 (4)	0.066 (4)	−0.016 (3)	0.016 (3)	0.020 (3)
O3A4	0.118 (5)	0.056 (4)	0.063 (4)	−0.024 (4)	0.000 (3)	0.002 (3)
O4A4	0.188 (6)	0.072 (4)	0.083 (4)	−0.001 (5)	0.088 (4)	−0.012 (4)
O5A4	0.063 (4)	0.058 (4)	0.049 (3)	0.005 (3)	0.010 (3)	0.002 (3)
C1B4	0.072 (6)	0.061 (6)	0.057 (5)	0.001 (5)	0.007 (5)	−0.012 (5)
C2B4	0.071 (6)	0.032 (5)	0.054 (5)	0.009 (5)	0.020 (4)	0.005 (4)
C3B4	0.059 (5)	0.062 (6)	0.041 (5)	−0.003 (5)	0.025 (4)	−0.013 (4)
C4B4	0.075 (6)	0.036 (5)	0.052 (5)	−0.004 (5)	0.014 (5)	−0.004 (4)
C5B4	0.064 (6)	0.032 (6)	0.089 (7)	−0.001 (5)	0.013 (5)	0.004 (5)
C6B4	0.136 (9)	0.085 (8)	0.105 (8)	−0.009 (7)	0.056 (7)	0.021 (6)
C7B4	0.099 (9)	0.121 (10)	0.138 (9)	−0.031 (8)	−0.036 (7)	−0.034 (8)
O7B4	0.120 (5)	0.069 (4)	0.067 (4)	−0.012 (4)	−0.004 (4)	−0.016 (4)
O2B4	0.091 (4)	0.033 (3)	0.050 (3)	−0.012 (3)	0.019 (3)	−0.002 (3)
O3B4	0.075 (4)	0.049 (4)	0.071 (4)	−0.002 (3)	0.023 (3)	−0.012 (3)
O4B4	0.086 (4)	0.049 (4)	0.090 (4)	0.001 (3)	0.028 (4)	0.009 (3)
O5B4	0.066 (4)	0.074 (5)	0.084 (4)	0.000 (3)	0.025 (3)	−0.017 (4)
O1	0.243 (9)	0.089 (6)	0.114 (6)	−0.010 (6)	0.057 (6)	−0.003 (5)
O2	0.191 (7)	0.070 (5)	0.085 (4)	0.014 (5)	0.037 (5)	−0.014 (4)
O3	0.223 (8)	0.116 (6)	0.060 (4)	0.022 (6)	0.047 (4)	0.003 (4)
O4	0.185 (10)	0.263 (13)	0.283 (13)	−0.015 (10)	0.038 (9)	0.057 (12)

### Geometric parameters (Å, º)

**Table:**

C1A1—O2B1	1.403 (8)	C2A3—O2A3	1.438 (8)
C1A1—O5A1	1.420 (8)	C2A3—C3A3	1.499 (10)
C1A1—C2A1	1.499 (10)	C2A3—H2A5	1.0000
C1A1—H1A1	1.0000	C3A3—O3A3	1.413 (9)
C2A1—O2A1	1.429 (8)	C3A3—C4A3	1.478 (9)
C2A1—C3A1	1.472 (11)	C3A3—H3A5	1.0000
C2A1—H2A1	1.0000	C4A3—O4A3	1.449 (9)
C3A1—O3A1	1.424 (9)	C4A3—C5A3	1.520 (10)
C3A1—C4A1	1.521 (11)	C4A3—H4A5	1.0000
C3A1—H3A1	1.0000	C5A3—O5A3	1.421 (9)
C4A1—O4A1	1.427 (9)	C5A3—C6A3	1.512 (10)
C4A1—C5A1	1.512 (11)	C5A3—H5A3	1.0000
C4A1—H4A1	1.0000	C6A3—H6A7	0.9800
C5A1—O5A1	1.435 (9)	C6A3—H6A8	0.9800
C5A1—C6A1	1.497 (10)	C6A3—H6A9	0.9800
C5A1—H5A1	1.0000	O2A3—H2A6	0.8400
C6A1—H6A1	0.9800	O3A3—H3A6	0.8400
C6A1—H6A2	0.9800	O4A3—H4A6	0.8400
C6A1—H6A3	0.9800	C1B3—O5B3	1.379 (10)
O2A1—H2A2	0.8400	C1B3—O7B3	1.419 (10)
O3A1—H3A2	0.8400	C1B3—C2B3	1.521 (10)
O4A1—H4A2	0.8400	C1B3—H1B3	1.0000
C1B1—O7B1	1.395 (10)	C2B3—O2B3	1.470 (8)
C1B1—O5B1	1.396 (10)	C2B3—C3B3	1.509 (10)
C1B1—C2B1	1.549 (10)	C2B3—H2B3	1.0000
C1B1—H1B1	1.0000	C3B3—O3B3	1.410 (8)
C2B1—O2B1	1.450 (8)	C3B3—C4B3	1.480 (10)
C2B1—C3B1	1.498 (10)	C3B3—H3B5	1.0000
C2B1—H2B1	1.0000	C4B3—O4B3	1.432 (8)
C3B1—O3B1	1.405 (8)	C4B3—C5B3	1.497 (10)
C3B1—C4B1	1.506 (10)	C4B3—H4B5	1.0000
C3B1—H3B1	1.0000	C5B3—O5B3	1.452 (9)
C4B1—O4B1	1.439 (8)	C5B3—C6B3	1.518 (12)
C4B1—C5B1	1.537 (10)	C5B3—H5B3	1.0000
C4B1—H4B1	1.0000	C6B3—H6B7	0.9800
C5B1—O5B1	1.416 (9)	C6B3—H6B8	0.9800
C5B1—C6B1	1.531 (11)	C6B3—H6B9	0.9800
C5B1—H5B1	1.0000	C7B3—O7B3	1.423 (11)
C6B1—H6B1	0.9800	C7B3—H7B7	0.9800
C6B1—H6B2	0.9800	C7B3—H7B8	0.9800
C6B1—H6B3	0.9800	C7B3—H7B9	0.9800
C7B1—O7B1	1.383 (10)	O3B3—H3B6	0.8400
C7B1—H7B1	0.9800	O4B3—H4B6	0.8400
C7B1—H7B2	0.9800	C1A4—O5A4	1.388 (8)
C7B1—H7B3	0.9800	C1A4—O2B4	1.432 (9)
O3B1—H3B2	0.8400	C1A4—C2A4	1.505 (10)
O4B1—H4B2	0.8400	C1A4—H1A4	1.0000
C1A2—O5A2	1.400 (9)	C2A4—O2A4	1.421 (9)
C1A2—O2B2	1.420 (8)	C2A4—C3A4	1.536 (10)
C1A2—C2A2	1.526 (10)	C2A4—H2A7	1.0000
C1A2—H1A2	1.0000	C3A4—O3A4	1.401 (9)
C2A2—O2A2	1.428 (9)	C3A4—C4A4	1.524 (11)
C2A2—C3A2	1.490 (10)	C3A4—H3A7	1.0000
C2A2—H2A3	1.0000	C4A4—O4A4	1.407 (9)
C3A2—O3A2	1.409 (8)	C4A4—C5A4	1.511 (11)
C3A2—C4A2	1.503 (10)	C4A4—H4A7	1.0000
C3A2—H3A3	1.0000	C5A4—O5A4	1.421 (9)
C4A2—O4A2	1.418 (8)	C5A4—C6A4	1.543 (11)
C4A2—C5A2	1.506 (10)	C5A4—H5A4	1.0000
C4A2—H4A3	1.0000	C6A4—H6AX	0.9800
C5A2—O5A2	1.426 (9)	C6A4—H6AY	0.9800
C5A2—C6A2	1.514 (11)	C6A4—H6AZ	0.9800
C5A2—H5A2	1.0000	O2A4—H2A8	0.8400
C6A2—H6A4	0.9800	O3A4—H3A8	0.8400
C6A2—H6A5	0.9800	O4A4—H4A8	0.8400
C6A2—H6A6	0.9800	C1B4—O7B4	1.383 (9)
O2A2—H2A4	0.8400	C1B4—O5B4	1.427 (9)
O3A2—H3A4	0.8400	C1B4—C2B4	1.512 (10)
O4A2—H4A4	0.8400	C1B4—H1B4	1.0000
C1B2—O7B2	1.405 (8)	C2B4—O2B4	1.421 (8)
C1B2—O5B2	1.430 (9)	C2B4—C3B4	1.491 (10)
C1B2—C2B2	1.546 (10)	C2B4—H2B4	1.0000
C1B2—H1B2	1.0000	C3B4—O3B4	1.415 (8)
C2B2—O2B2	1.431 (8)	C3B4—C4B4	1.467 (10)
C2B2—C3B2	1.465 (10)	C3B4—H3B7	1.0000
C2B2—H2B2	1.0000	C4B4—O4B4	1.423 (8)
C3B2—O3B2	1.443 (8)	C4B4—C5B4	1.501 (10)
C3B2—C4B2	1.477 (9)	C4B4—H4B7	1.0000
C3B2—H3B3	1.0000	C5B4—O5B4	1.452 (9)
C4B2—O4B2	1.428 (8)	C5B4—C6B4	1.505 (12)
C4B2—C5B2	1.517 (10)	C5B4—H5B4	1.0000
C4B2—H4B3	1.0000	C6B4—H6BX	0.9800
C5B2—O5B2	1.428 (10)	C6B4—H6BY	0.9800
C5B2—C6B2	1.484 (11)	C6B4—H6BZ	0.9800
C5B2—H5B2	1.0000	C7B4—O7B4	1.451 (10)
C6B2—H6B4	0.9800	C7B4—H7BX	0.9800
C6B2—H6B5	0.9800	C7B4—H7BY	0.9800
C6B2—H6B6	0.9800	C7B4—H7BZ	0.9800
C7B2—O7B2	1.402 (9)	O3B4—H3B8	0.8400
C7B2—H7B4	0.9800	O4B4—H4B8	0.8400
C7B2—H7B5	0.9800	O1—H11	0.873 (14)
C7B2—H7B6	0.9800	O1—H12	0.860 (14)
O3B2—H3B4	0.8400	O2—H21	0.850 (14)
O4B2—H4B4	0.8400	O2—H22	0.849 (14)
C1A3—O5A3	1.409 (8)	O3—H31	0.849 (14)
C1A3—O2B3	1.417 (8)	O3—H32	0.847 (14)
C1A3—C2A3	1.485 (10)	O4—H41	0.850 (14)
C1A3—H1A3	1.0000	O4—H42	0.854 (14)
			
O2B1—C1A1—O5A1	112.4 (6)	O2B3—C1A3—C2A3	107.3 (6)
O2B1—C1A1—C2A1	109.3 (6)	O5A3—C1A3—H1A3	108.7
O5A1—C1A1—C2A1	112.1 (6)	O2B3—C1A3—H1A3	108.7
O2B1—C1A1—H1A1	107.6	C2A3—C1A3—H1A3	108.7
O5A1—C1A1—H1A1	107.6	O2A3—C2A3—C1A3	107.2 (6)
C2A1—C1A1—H1A1	107.6	O2A3—C2A3—C3A3	109.1 (6)
O2A1—C2A1—C3A1	108.8 (6)	C1A3—C2A3—C3A3	113.2 (7)
O2A1—C2A1—C1A1	109.9 (7)	O2A3—C2A3—H2A5	109.1
C3A1—C2A1—C1A1	110.8 (7)	C1A3—C2A3—H2A5	109.1
O2A1—C2A1—H2A1	109.1	C3A3—C2A3—H2A5	109.1
C3A1—C2A1—H2A1	109.1	O3A3—C3A3—C4A3	110.6 (6)
C1A1—C2A1—H2A1	109.1	O3A3—C3A3—C2A3	110.9 (6)
O3A1—C3A1—C2A1	109.9 (7)	C4A3—C3A3—C2A3	111.2 (7)
O3A1—C3A1—C4A1	110.2 (7)	O3A3—C3A3—H3A5	108.0
C2A1—C3A1—C4A1	112.5 (7)	C4A3—C3A3—H3A5	108.0
O3A1—C3A1—H3A1	108.0	C2A3—C3A3—H3A5	108.0
C2A1—C3A1—H3A1	108.0	O4A3—C4A3—C3A3	111.6 (7)
C4A1—C3A1—H3A1	108.0	O4A3—C4A3—C5A3	107.7 (6)
O4A1—C4A1—C5A1	111.0 (7)	C3A3—C4A3—C5A3	112.1 (6)
O4A1—C4A1—C3A1	110.3 (7)	O4A3—C4A3—H4A5	108.4
C5A1—C4A1—C3A1	109.0 (7)	C3A3—C4A3—H4A5	108.4
O4A1—C4A1—H4A1	108.8	C5A3—C4A3—H4A5	108.4
C5A1—C4A1—H4A1	108.8	O5A3—C5A3—C6A3	105.6 (7)
C3A1—C4A1—H4A1	108.8	O5A3—C5A3—C4A3	111.2 (6)
O5A1—C5A1—C6A1	107.0 (7)	C6A3—C5A3—C4A3	117.8 (7)
O5A1—C5A1—C4A1	109.5 (6)	O5A3—C5A3—H5A3	107.3
C6A1—C5A1—C4A1	113.8 (7)	C6A3—C5A3—H5A3	107.3
O5A1—C5A1—H5A1	108.8	C4A3—C5A3—H5A3	107.3
C6A1—C5A1—H5A1	108.8	C5A3—C6A3—H6A7	109.5
C4A1—C5A1—H5A1	108.8	C5A3—C6A3—H6A8	109.5
C5A1—C6A1—H6A1	109.5	H6A7—C6A3—H6A8	109.5
C5A1—C6A1—H6A2	109.5	C5A3—C6A3—H6A9	109.5
H6A1—C6A1—H6A2	109.5	H6A7—C6A3—H6A9	109.5
C5A1—C6A1—H6A3	109.5	H6A8—C6A3—H6A9	109.5
H6A1—C6A1—H6A3	109.5	C2A3—O2A3—H2A6	109.5
H6A2—C6A1—H6A3	109.5	C3A3—O3A3—H3A6	109.5
C2A1—O2A1—H2A2	109.5	C4A3—O4A3—H4A6	109.5
C3A1—O3A1—H3A2	109.5	C1A3—O5A3—C5A3	114.8 (6)
C4A1—O4A1—H4A2	109.5	O5B3—C1B3—O7B3	111.6 (7)
C1A1—O5A1—C5A1	112.7 (6)	O5B3—C1B3—C2B3	113.2 (7)
O7B1—C1B1—O5B1	113.1 (7)	O7B3—C1B3—C2B3	104.2 (7)
O7B1—C1B1—C2B1	105.2 (7)	O5B3—C1B3—H1B3	109.2
O5B1—C1B1—C2B1	112.5 (7)	O7B3—C1B3—H1B3	109.2
O7B1—C1B1—H1B1	108.6	C2B3—C1B3—H1B3	109.2
O5B1—C1B1—H1B1	108.6	O2B3—C2B3—C3B3	106.8 (6)
C2B1—C1B1—H1B1	108.6	O2B3—C2B3—C1B3	108.4 (6)
O2B1—C2B1—C3B1	106.4 (6)	C3B3—C2B3—C1B3	114.0 (7)
O2B1—C2B1—C1B1	108.8 (6)	O2B3—C2B3—H2B3	109.2
C3B1—C2B1—C1B1	112.2 (6)	C3B3—C2B3—H2B3	109.2
O2B1—C2B1—H2B1	109.8	C1B3—C2B3—H2B3	109.2
C3B1—C2B1—H2B1	109.8	O3B3—C3B3—C4B3	112.4 (7)
C1B1—C2B1—H2B1	109.8	O3B3—C3B3—C2B3	113.6 (6)
O3B1—C3B1—C2B1	111.6 (6)	C4B3—C3B3—C2B3	111.8 (6)
O3B1—C3B1—C4B1	110.9 (6)	O3B3—C3B3—H3B5	106.1
C2B1—C3B1—C4B1	111.6 (6)	C4B3—C3B3—H3B5	106.1
O3B1—C3B1—H3B1	107.5	C2B3—C3B3—H3B5	106.1
C2B1—C3B1—H3B1	107.5	O4B3—C4B3—C3B3	110.3 (6)
C4B1—C3B1—H3B1	107.5	O4B3—C4B3—C5B3	107.8 (6)
O4B1—C4B1—C3B1	108.8 (6)	C3B3—C4B3—C5B3	111.3 (7)
O4B1—C4B1—C5B1	110.3 (6)	O4B3—C4B3—H4B5	109.1
C3B1—C4B1—C5B1	109.7 (7)	C3B3—C4B3—H4B5	109.1
O4B1—C4B1—H4B1	109.4	C5B3—C4B3—H4B5	109.1
C3B1—C4B1—H4B1	109.4	O5B3—C5B3—C4B3	107.7 (6)
C5B1—C4B1—H4B1	109.4	O5B3—C5B3—C6B3	109.0 (7)
O5B1—C5B1—C6B1	109.4 (7)	C4B3—C5B3—C6B3	114.5 (8)
O5B1—C5B1—C4B1	107.9 (6)	O5B3—C5B3—H5B3	108.5
C6B1—C5B1—C4B1	111.8 (7)	C4B3—C5B3—H5B3	108.5
O5B1—C5B1—H5B1	109.3	C6B3—C5B3—H5B3	108.5
C6B1—C5B1—H5B1	109.3	C5B3—C6B3—H6B7	109.5
C4B1—C5B1—H5B1	109.3	C5B3—C6B3—H6B8	109.5
C5B1—C6B1—H6B1	109.5	H6B7—C6B3—H6B8	109.5
C5B1—C6B1—H6B2	109.5	C5B3—C6B3—H6B9	109.5
H6B1—C6B1—H6B2	109.5	H6B7—C6B3—H6B9	109.5
C5B1—C6B1—H6B3	109.5	H6B8—C6B3—H6B9	109.5
H6B1—C6B1—H6B3	109.5	O7B3—C7B3—H7B7	109.5
H6B2—C6B1—H6B3	109.5	O7B3—C7B3—H7B8	109.5
O7B1—C7B1—H7B1	109.5	H7B7—C7B3—H7B8	109.5
O7B1—C7B1—H7B2	109.5	O7B3—C7B3—H7B9	109.5
H7B1—C7B1—H7B2	109.5	H7B7—C7B3—H7B9	109.5
O7B1—C7B1—H7B3	109.5	H7B8—C7B3—H7B9	109.5
H7B1—C7B1—H7B3	109.5	C1B3—O7B3—C7B3	111.1 (8)
H7B2—C7B1—H7B3	109.5	C1A3—O2B3—C2B3	114.2 (6)
C7B1—O7B1—C1B1	113.4 (8)	C3B3—O3B3—H3B6	109.5
C1A1—O2B1—C2B1	113.9 (6)	C4B3—O4B3—H4B6	109.5
C3B1—O3B1—H3B2	109.5	C1B3—O5B3—C5B3	115.1 (7)
C4B1—O4B1—H4B2	109.5	O5A4—C1A4—O2B4	111.5 (6)
C1B1—O5B1—C5B1	115.2 (7)	O5A4—C1A4—C2A4	112.6 (7)
O5A2—C1A2—O2B2	111.8 (6)	O2B4—C1A4—C2A4	107.4 (6)
O5A2—C1A2—C2A2	112.8 (6)	O5A4—C1A4—H1A4	108.4
O2B2—C1A2—C2A2	105.6 (6)	O2B4—C1A4—H1A4	108.4
O5A2—C1A2—H1A2	108.8	C2A4—C1A4—H1A4	108.4
O2B2—C1A2—H1A2	108.8	O2A4—C2A4—C1A4	110.9 (6)
C2A2—C1A2—H1A2	108.8	O2A4—C2A4—C3A4	111.0 (6)
O2A2—C2A2—C3A2	110.5 (6)	C1A4—C2A4—C3A4	109.4 (7)
O2A2—C2A2—C1A2	109.3 (6)	O2A4—C2A4—H2A7	108.5
C3A2—C2A2—C1A2	109.1 (6)	C1A4—C2A4—H2A7	108.5
O2A2—C2A2—H2A3	109.3	C3A4—C2A4—H2A7	108.5
C3A2—C2A2—H2A3	109.3	O3A4—C3A4—C4A4	113.0 (7)
C1A2—C2A2—H2A3	109.3	O3A4—C3A4—C2A4	109.0 (7)
O3A2—C3A2—C2A2	108.0 (6)	C4A4—C3A4—C2A4	112.2 (6)
O3A2—C3A2—C4A2	112.7 (7)	O3A4—C3A4—H3A7	107.5
C2A2—C3A2—C4A2	112.5 (7)	C4A4—C3A4—H3A7	107.5
O3A2—C3A2—H3A3	107.8	C2A4—C3A4—H3A7	107.5
C2A2—C3A2—H3A3	107.8	O4A4—C4A4—C5A4	109.8 (7)
C4A2—C3A2—H3A3	107.8	O4A4—C4A4—C3A4	110.2 (7)
O4A2—C4A2—C3A2	109.4 (7)	C5A4—C4A4—C3A4	109.4 (7)
O4A2—C4A2—C5A2	110.9 (6)	O4A4—C4A4—H4A7	109.1
C3A2—C4A2—C5A2	112.6 (7)	C5A4—C4A4—H4A7	109.1
O4A2—C4A2—H4A3	107.9	C3A4—C4A4—H4A7	109.1
C3A2—C4A2—H4A3	107.9	O5A4—C5A4—C4A4	112.2 (7)
C5A2—C4A2—H4A3	107.9	O5A4—C5A4—C6A4	107.9 (7)
O5A2—C5A2—C4A2	110.8 (6)	C4A4—C5A4—C6A4	110.1 (8)
O5A2—C5A2—C6A2	106.8 (7)	O5A4—C5A4—H5A4	108.9
C4A2—C5A2—C6A2	112.3 (8)	C4A4—C5A4—H5A4	108.9
O5A2—C5A2—H5A2	109.0	C6A4—C5A4—H5A4	108.9
C4A2—C5A2—H5A2	109.0	C5A4—C6A4—H6AX	109.5
C6A2—C5A2—H5A2	109.0	C5A4—C6A4—H6AY	109.5
C5A2—C6A2—H6A4	109.5	H6AX—C6A4—H6AY	109.5
C5A2—C6A2—H6A5	109.5	C5A4—C6A4—H6AZ	109.5
H6A4—C6A2—H6A5	109.5	H6AX—C6A4—H6AZ	109.5
C5A2—C6A2—H6A6	109.5	H6AY—C6A4—H6AZ	109.5
H6A4—C6A2—H6A6	109.5	C2A4—O2A4—H2A8	109.5
H6A5—C6A2—H6A6	109.5	C3A4—O3A4—H3A8	109.5
C2A2—O2A2—H2A4	109.5	C4A4—O4A4—H4A8	109.5
C3A2—O3A2—H3A4	109.5	C1A4—O5A4—C5A4	113.4 (6)
C4A2—O4A2—H4A4	109.5	O7B4—C1B4—O5B4	111.9 (7)
C1A2—O5A2—C5A2	113.7 (6)	O7B4—C1B4—C2B4	109.2 (7)
O7B2—C1B2—O5B2	111.0 (6)	O5B4—C1B4—C2B4	110.6 (6)
O7B2—C1B2—C2B2	106.4 (6)	O7B4—C1B4—H1B4	108.4
O5B2—C1B2—C2B2	110.1 (6)	O5B4—C1B4—H1B4	108.4
O7B2—C1B2—H1B2	109.8	C2B4—C1B4—H1B4	108.4
O5B2—C1B2—H1B2	109.8	O2B4—C2B4—C3B4	107.3 (6)
C2B2—C1B2—H1B2	109.8	O2B4—C2B4—C1B4	109.7 (6)
O2B2—C2B2—C3B2	108.7 (6)	C3B4—C2B4—C1B4	112.7 (7)
O2B2—C2B2—C1B2	109.0 (6)	O2B4—C2B4—H2B4	109.0
C3B2—C2B2—C1B2	111.8 (7)	C3B4—C2B4—H2B4	109.0
O2B2—C2B2—H2B2	109.1	C1B4—C2B4—H2B4	109.0
C3B2—C2B2—H2B2	109.1	O3B4—C3B4—C4B4	112.6 (6)
C1B2—C2B2—H2B2	109.1	O3B4—C3B4—C2B4	111.3 (7)
O3B2—C3B2—C2B2	109.3 (6)	C4B4—C3B4—C2B4	112.3 (7)
O3B2—C3B2—C4B2	110.3 (6)	O3B4—C3B4—H3B7	106.7
C2B2—C3B2—C4B2	112.7 (6)	C4B4—C3B4—H3B7	106.7
O3B2—C3B2—H3B3	108.2	C2B4—C3B4—H3B7	106.7
C2B2—C3B2—H3B3	108.2	O4B4—C4B4—C3B4	111.9 (7)
C4B2—C3B2—H3B3	108.2	O4B4—C4B4—C5B4	107.7 (6)
O4B2—C4B2—C3B2	109.8 (6)	C3B4—C4B4—C5B4	110.9 (7)
O4B2—C4B2—C5B2	108.2 (7)	O4B4—C4B4—H4B7	108.8
C3B2—C4B2—C5B2	108.7 (7)	C3B4—C4B4—H4B7	108.8
O4B2—C4B2—H4B3	110.0	C5B4—C4B4—H4B7	108.8
C3B2—C4B2—H4B3	110.0	O5B4—C5B4—C4B4	106.1 (6)
C5B2—C4B2—H4B3	110.0	O5B4—C5B4—C6B4	106.2 (7)
O5B2—C5B2—C6B2	108.0 (7)	C4B4—C5B4—C6B4	117.0 (8)
O5B2—C5B2—C4B2	107.8 (7)	O5B4—C5B4—H5B4	109.1
C6B2—C5B2—C4B2	114.7 (8)	C4B4—C5B4—H5B4	109.1
O5B2—C5B2—H5B2	108.7	C6B4—C5B4—H5B4	109.1
C6B2—C5B2—H5B2	108.7	C5B4—C6B4—H6BX	109.5
C4B2—C5B2—H5B2	108.7	C5B4—C6B4—H6BY	109.5
C5B2—C6B2—H6B4	109.5	H6BX—C6B4—H6BY	109.5
C5B2—C6B2—H6B5	109.5	C5B4—C6B4—H6BZ	109.5
H6B4—C6B2—H6B5	109.5	H6BX—C6B4—H6BZ	109.5
C5B2—C6B2—H6B6	109.5	H6BY—C6B4—H6BZ	109.5
H6B4—C6B2—H6B6	109.5	O7B4—C7B4—H7BX	109.5
H6B5—C6B2—H6B6	109.5	O7B4—C7B4—H7BY	109.5
O7B2—C7B2—H7B4	109.5	H7BX—C7B4—H7BY	109.5
O7B2—C7B2—H7B5	109.5	O7B4—C7B4—H7BZ	109.5
H7B4—C7B2—H7B5	109.5	H7BX—C7B4—H7BZ	109.5
O7B2—C7B2—H7B6	109.5	H7BY—C7B4—H7BZ	109.5
H7B4—C7B2—H7B6	109.5	C1B4—O7B4—C7B4	111.8 (7)
H7B5—C7B2—H7B6	109.5	C2B4—O2B4—C1A4	116.2 (5)
C7B2—O7B2—C1B2	111.4 (7)	C3B4—O3B4—H3B8	109.5
C1A2—O2B2—C2B2	117.2 (6)	C4B4—O4B4—H4B8	109.5
C3B2—O3B2—H3B4	109.5	C1B4—O5B4—C5B4	114.6 (6)
C4B2—O4B2—H4B4	109.5	H11—O1—H12	102 (2)
C5B2—O5B2—C1B2	116.3 (6)	H21—O2—H22	104 (2)
O5A3—C1A3—O2B3	111.3 (6)	H31—O3—H32	105 (2)
O5A3—C1A3—C2A3	112.0 (6)	H41—O4—H42	105 (2)
			
O2B1—C1A1—C2A1—O2A1	−166.8 (6)	O5A3—C1A3—C2A3—O2A3	69.4 (7)
O5A1—C1A1—C2A1—O2A1	67.9 (9)	O2B3—C1A3—C2A3—O2A3	−168.2 (5)
O2B1—C1A1—C2A1—C3A1	72.8 (8)	O5A3—C1A3—C2A3—C3A3	−51.0 (8)
O5A1—C1A1—C2A1—C3A1	−52.4 (9)	O2B3—C1A3—C2A3—C3A3	71.4 (7)
O2A1—C2A1—C3A1—O3A1	52.9 (9)	O2A3—C2A3—C3A3—O3A3	53.3 (8)
C1A1—C2A1—C3A1—O3A1	173.9 (7)	C1A3—C2A3—C3A3—O3A3	172.6 (6)
O2A1—C2A1—C3A1—C4A1	−70.3 (8)	O2A3—C2A3—C3A3—C4A3	−70.2 (8)
C1A1—C2A1—C3A1—C4A1	50.7 (9)	C1A3—C2A3—C3A3—C4A3	49.1 (8)
O3A1—C3A1—C4A1—O4A1	61.3 (8)	O3A3—C3A3—C4A3—O4A3	66.1 (8)
C2A1—C3A1—C4A1—O4A1	−175.6 (6)	C2A3—C3A3—C4A3—O4A3	−170.2 (6)
O3A1—C3A1—C4A1—C5A1	−176.5 (7)	O3A3—C3A3—C4A3—C5A3	−173.0 (6)
C2A1—C3A1—C4A1—C5A1	−53.4 (9)	C2A3—C3A3—C4A3—C5A3	−49.3 (9)
O4A1—C4A1—C5A1—O5A1	178.6 (6)	O4A3—C4A3—C5A3—O5A3	175.3 (6)
C3A1—C4A1—C5A1—O5A1	56.8 (8)	C3A3—C4A3—C5A3—O5A3	52.1 (9)
O4A1—C4A1—C5A1—C6A1	−61.7 (10)	O4A3—C4A3—C5A3—C6A3	−62.7 (9)
C3A1—C4A1—C5A1—C6A1	176.5 (7)	C3A3—C4A3—C5A3—C6A3	174.1 (8)
O2B1—C1A1—O5A1—C5A1	−64.7 (7)	O2B3—C1A3—O5A3—C5A3	−65.1 (8)
C2A1—C1A1—O5A1—C5A1	58.8 (8)	C2A3—C1A3—O5A3—C5A3	55.0 (8)
C6A1—C5A1—O5A1—C1A1	175.0 (6)	C6A3—C5A3—O5A3—C1A3	175.9 (6)
C4A1—C5A1—O5A1—C1A1	−61.2 (8)	C4A3—C5A3—O5A3—C1A3	−55.3 (8)
O7B1—C1B1—C2B1—O2B1	−164.4 (6)	O5B3—C1B3—C2B3—O2B3	77.3 (8)
O5B1—C1B1—C2B1—O2B1	72.1 (8)	O7B3—C1B3—C2B3—O2B3	−161.3 (6)
O7B1—C1B1—C2B1—C3B1	78.2 (8)	O5B3—C1B3—C2B3—C3B3	−41.5 (10)
O5B1—C1B1—C2B1—C3B1	−45.4 (9)	O7B3—C1B3—C2B3—C3B3	80.0 (9)
O2B1—C2B1—C3B1—O3B1	52.2 (8)	O2B3—C2B3—C3B3—O3B3	50.5 (8)
C1B1—C2B1—C3B1—O3B1	171.1 (6)	C1B3—C2B3—C3B3—O3B3	170.1 (7)
O2B1—C2B1—C3B1—C4B1	−72.5 (7)	O2B3—C2B3—C3B3—C4B3	−78.1 (8)
C1B1—C2B1—C3B1—C4B1	46.3 (8)	C1B3—C2B3—C3B3—C4B3	41.6 (9)
O3B1—C3B1—C4B1—O4B1	59.8 (7)	O3B3—C3B3—C4B3—O4B3	59.3 (8)
C2B1—C3B1—C4B1—O4B1	−175.1 (6)	C2B3—C3B3—C4B3—O4B3	−171.5 (6)
O3B1—C3B1—C4B1—C5B1	−179.5 (6)	O3B3—C3B3—C4B3—C5B3	178.9 (6)
C2B1—C3B1—C4B1—C5B1	−54.4 (8)	C2B3—C3B3—C4B3—C5B3	−51.9 (8)
O4B1—C4B1—C5B1—O5B1	−179.8 (6)	O4B3—C4B3—C5B3—O5B3	−178.7 (7)
C3B1—C4B1—C5B1—O5B1	60.4 (8)	C3B3—C4B3—C5B3—O5B3	60.2 (9)
O4B1—C4B1—C5B1—C6B1	−59.4 (9)	O4B3—C4B3—C5B3—C6B3	−57.4 (9)
C3B1—C4B1—C5B1—C6B1	−179.2 (7)	C3B3—C4B3—C5B3—C6B3	−178.5 (7)
O5B1—C1B1—O7B1—C7B1	−71.8 (9)	O5B3—C1B3—O7B3—C7B3	−70.1 (9)
C2B1—C1B1—O7B1—C7B1	165.1 (7)	C2B3—C1B3—O7B3—C7B3	167.3 (8)
O5A1—C1A1—O2B1—C2B1	−78.6 (8)	O5A3—C1A3—O2B3—C2B3	−83.7 (7)
C2A1—C1A1—O2B1—C2B1	156.3 (6)	C2A3—C1A3—O2B3—C2B3	153.5 (6)
C3B1—C2B1—O2B1—C1A1	−151.5 (6)	C3B3—C2B3—O2B3—C1A3	−149.4 (6)
C1B1—C2B1—O2B1—C1A1	87.4 (7)	C1B3—C2B3—O2B3—C1A3	87.4 (8)
O7B1—C1B1—O5B1—C5B1	−63.7 (9)	O7B3—C1B3—O5B3—C5B3	−64.5 (9)
C2B1—C1B1—O5B1—C5B1	55.4 (8)	C2B3—C1B3—O5B3—C5B3	52.7 (9)
C6B1—C5B1—O5B1—C1B1	175.6 (6)	C4B3—C5B3—O5B3—C1B3	−61.9 (9)
C4B1—C5B1—O5B1—C1B1	−62.6 (8)	C6B3—C5B3—O5B3—C1B3	173.3 (7)
O5A2—C1A2—C2A2—O2A2	66.6 (8)	O5A4—C1A4—C2A4—O2A4	68.7 (8)
O2B2—C1A2—C2A2—O2A2	−171.0 (6)	O2B4—C1A4—C2A4—O2A4	−168.2 (6)
O5A2—C1A2—C2A2—C3A2	−54.3 (8)	O5A4—C1A4—C2A4—C3A4	−54.0 (8)
O2B2—C1A2—C2A2—C3A2	68.1 (8)	O2B4—C1A4—C2A4—C3A4	69.1 (8)
O2A2—C2A2—C3A2—O3A2	54.3 (8)	O2A4—C2A4—C3A4—O3A4	53.1 (8)
C1A2—C2A2—C3A2—O3A2	174.5 (6)	C1A4—C2A4—C3A4—O3A4	175.8 (6)
O2A2—C2A2—C3A2—C4A2	−70.7 (8)	O2A4—C2A4—C3A4—C4A4	−72.9 (8)
C1A2—C2A2—C3A2—C4A2	49.5 (9)	C1A4—C2A4—C3A4—C4A4	49.8 (9)
O3A2—C3A2—C4A2—O4A2	64.1 (8)	O3A4—C3A4—C4A4—O4A4	65.7 (9)
C2A2—C3A2—C4A2—O4A2	−173.4 (7)	C2A4—C3A4—C4A4—O4A4	−170.6 (7)
O3A2—C3A2—C4A2—C5A2	−172.1 (6)	O3A4—C3A4—C4A4—C5A4	−173.5 (6)
C2A2—C3A2—C4A2—C5A2	−49.6 (9)	C2A4—C3A4—C4A4—C5A4	−49.8 (9)
O4A2—C4A2—C5A2—O5A2	173.8 (6)	O4A4—C4A4—C5A4—O5A4	174.4 (7)
C3A2—C4A2—C5A2—O5A2	50.8 (9)	C3A4—C4A4—C5A4—O5A4	53.3 (9)
O4A2—C4A2—C5A2—C6A2	−66.9 (9)	O4A4—C4A4—C5A4—C6A4	−65.4 (9)
C3A2—C4A2—C5A2—C6A2	170.1 (7)	C3A4—C4A4—C5A4—C6A4	173.5 (7)
O2B2—C1A2—O5A2—C5A2	−59.7 (8)	O2B4—C1A4—O5A4—C5A4	−60.8 (8)
C2A2—C1A2—O5A2—C5A2	59.2 (8)	C2A4—C1A4—O5A4—C5A4	60.0 (8)
C4A2—C5A2—O5A2—C1A2	−56.3 (8)	C4A4—C5A4—O5A4—C1A4	−59.7 (9)
C6A2—C5A2—O5A2—C1A2	−178.9 (7)	C6A4—C5A4—O5A4—C1A4	178.8 (7)
O7B2—C1B2—C2B2—O2B2	−165.9 (6)	O7B4—C1B4—C2B4—O2B4	−163.3 (6)
O5B2—C1B2—C2B2—O2B2	73.8 (8)	O5B4—C1B4—C2B4—O2B4	73.2 (8)
O7B2—C1B2—C2B2—C3B2	73.9 (8)	O7B4—C1B4—C2B4—C3B4	77.2 (8)
O5B2—C1B2—C2B2—C3B2	−46.4 (9)	O5B4—C1B4—C2B4—C3B4	−46.3 (9)
O2B2—C2B2—C3B2—O3B2	54.2 (7)	O2B4—C2B4—C3B4—O3B4	53.5 (8)
C1B2—C2B2—C3B2—O3B2	174.5 (6)	C1B4—C2B4—C3B4—O3B4	174.4 (6)
O2B2—C2B2—C3B2—C4B2	−68.8 (8)	O2B4—C2B4—C3B4—C4B4	−73.7 (8)
C1B2—C2B2—C3B2—C4B2	51.6 (9)	C1B4—C2B4—C3B4—C4B4	47.2 (9)
O3B2—C3B2—C4B2—O4B2	61.0 (8)	O3B4—C3B4—C4B4—O4B4	57.9 (9)
C2B2—C3B2—C4B2—O4B2	−176.5 (6)	C2B4—C3B4—C4B4—O4B4	−175.6 (6)
O3B2—C3B2—C4B2—C5B2	179.2 (6)	O3B4—C3B4—C4B4—C5B4	178.2 (6)
C2B2—C3B2—C4B2—C5B2	−58.4 (9)	C2B4—C3B4—C4B4—C5B4	−55.3 (9)
O4B2—C4B2—C5B2—O5B2	179.1 (6)	O4B4—C4B4—C5B4—O5B4	−176.5 (6)
C3B2—C4B2—C5B2—O5B2	59.9 (8)	C3B4—C4B4—C5B4—O5B4	60.8 (9)
O4B2—C4B2—C5B2—C6B2	−60.5 (10)	O4B4—C4B4—C5B4—C6B4	−58.3 (9)
C3B2—C4B2—C5B2—C6B2	−179.7 (8)	C3B4—C4B4—C5B4—C6B4	179.0 (7)
O5B2—C1B2—O7B2—C7B2	−69.7 (8)	O5B4—C1B4—O7B4—C7B4	−69.1 (9)
C2B2—C1B2—O7B2—C7B2	170.6 (7)	C2B4—C1B4—O7B4—C7B4	168.2 (7)
O5A2—C1A2—O2B2—C2B2	−90.7 (7)	C3B4—C2B4—O2B4—C1A4	−150.0 (6)
C2A2—C1A2—O2B2—C2B2	146.2 (6)	C1B4—C2B4—O2B4—C1A4	87.2 (8)
C3B2—C2B2—O2B2—C1A2	−153.8 (6)	O5A4—C1A4—O2B4—C2B4	−82.4 (8)
C1B2—C2B2—O2B2—C1A2	84.1 (7)	C2A4—C1A4—O2B4—C2B4	153.8 (6)
C6B2—C5B2—O5B2—C1B2	174.9 (7)	O7B4—C1B4—O5B4—C5B4	−65.3 (8)
C4B2—C5B2—O5B2—C1B2	−60.5 (9)	C2B4—C1B4—O5B4—C5B4	56.6 (8)
O7B2—C1B2—O5B2—C5B2	−64.2 (8)	C4B4—C5B4—O5B4—C1B4	−63.2 (8)
C2B2—C1B2—O5B2—C5B2	53.3 (9)	C6B4—C5B4—O5B4—C1B4	171.6 (7)

### Hydrogen-bond geometry (Å, º)

**Table:**

*D*—H···*A*	*D*—H	H···*A*	*D*···*A*	*D*—H···*A*
O2*A*1—H2*A*2···O2	0.84	1.96	2.770 (9)	161
O3*A*1—H3*A*2···O4*A*2	0.84	2.59	3.366 (10)	154
O3*B*1—H3*B*2···O3*A*3^i^	0.84	1.95	2.766 (8)	164
O4*B*1—H4*B*2···O1^ii^	0.84	1.89	2.693 (10)	158
O2*A*2—H2*A*4···O1^iii^	0.84	2.04	2.834 (10)	159
O3*A*2—H3*A*4···O3^ii^	0.84	1.85	2.647 (9)	159
O4*A*2—H4*A*4···O4*A*1	0.84	2.09	2.862 (8)	152
O3*B*2—H3*B*4···O3*A*2^iv^	0.84	2.06	2.710 (7)	133
O4*B*2—H4*B*4···O2	0.84	2.20	2.952 (8)	150
O2*A*3—H2*A*6···O4*B*1^ii^	0.84	1.98	2.791 (8)	161
O3*A*3—H3*A*6···O3^ii^	0.84	1.92	2.741 (9)	165
O4*A*3—H4*A*6···O4*A*4	0.84	2.13	2.731 (9)	128
O3*B*3—H3*B*6···O2*A*1^iii^	0.84	2.58	3.309 (8)	146
O3*B*3—H3*B*6···O3*A*1^iii^	0.84	2.13	2.855 (8)	145
O4*B*3—H4*B*6···O2*A*1^iii^	0.84	2.00	2.754 (9)	149
C3*A*4—H3*A*7···O3*A*3	1.00	2.56	3.431 (10)	146
O3*A*4—H3*A*8···O3*B*4^i^	0.84	2.08	2.761 (8)	138
O4*A*4—H4*A*8···O4*A*1	0.84	2.05	2.717 (9)	136
O3*B*4—H3*B*8···O3*B*1^ii^	0.84	2.02	2.843 (7)	168
O4*B*4—H4*B*8···O2*A*4^ii^	0.84	2.08	2.859 (8)	155
O1—H12···O4*B*3^iv^	0.86 (1)	1.86 (2)	2.718 (10)	174 (7)
O2—H21···O4*B*4^v^	0.85 (1)	2.29 (4)	3.030 (9)	145 (6)
O2—H22···O5*A*3^v^	0.85 (1)	2.62 (2)	3.461 (9)	169 (5)
O3—H31···O3*A*4^i^	0.85 (1)	2.00 (4)	2.695 (8)	139 (5)
O3—H32···O4	0.85 (1)	1.80 (3)	2.582 (13)	152 (7)
O4—H41···O7*B*1	0.85 (1)	2.29 (3)	3.037 (14)	147 (5)
O4—H42···O7*B*3^vi^	0.85 (1)	2.26 (3)	3.021 (14)	148 (4)

Symmetry codes: (i) −*x*, *y*−1/2, −*z*+1; (ii) −*x*, *y*+1/2, −*z*+1; (iii) −*x*, *y*+1/2, −*z*; (iv) −*x*, *y*−1/2, −*z*; (v) *x*, *y*−1, *z*; (vi) *x*, *y*−1, *z*+1.
